# Draft genome sequence of *Geobacillus* sp. strain G4 isolated from the Calientes geothermal field in Tacna, Peru

**DOI:** 10.1128/mra.00717-24

**Published:** 2024-11-21

**Authors:** Roberto Castellanos, Karita C. R. Santos, Alonso R. Poma Ticona, Heber E. Ramirez-Arua, Soledad A. Bornás-Acosta, Jéssica Pinheiro Silva, Pedro R. Vieira Hamann, Fabyano A. C. Lopes

**Affiliations:** 1 Enzyme Biotechnology Research Laboratory, Science Faculty, Universidad Nacional Jorge Basadre Grohmann, Tacna, Peru; 2 Laboratório de Microbiologia, Universidade Federal Do Tocantins, Porto Nacional, Brazil; 3 Laboratorio de Biología Molecular, Facultad de Farmacia y Bioquímica, Universidad Nacional Mayor de San Marcos, Lima, Peru; 4 São Carlos Institute of Physics, University of São Paulo, São Carlos, Brazil; SUNY College of Environmental Science and Forestry, Syracuse, New York, USA

**Keywords:** *Geobacillus*, thermophilic bacteria, geothermal spring, Calientes geothermal field

## Abstract

We report the draft genome sequence of *Geobacillus* sp. strain G4, isolated from a hot spring in the Calientes geothermal field, Tacna, Peru. The genome was assembled into 164 contigs, totaling 3,362,411 bp, with an N50 value of 236,119 bp and a guanine-cytosine (GC) content of 52.59%.

## ANNOUNCEMENT

*Geobacillus* sp. strain G4, a thermophilic bacterium, was isolated from sediment samples (upper 15 cm layer) collected from a geothermal spring in the Calientes geothermal field, Candarave, Tacna, Peru (UTM 0.379908 E, 81.09185 N). A total of 250 g of sediment was collected in sterile containers, and 10 g was enriched and then plated on Luria-Bertani (LB) medium at 60°C for 48 hours (1). Strain G4 was isolated and confirmed as axenic after three transfers. Strain G4 showed high amylolytic activity, determined by the Miller method (2): 100 µL of starch (0.5% wt/vol) and 100 µL of G4 culture supernatant were incubated at 60°C for 15 minutes; 300 µL of DNS reagent was added, and absorbance was read at 540 nm.

*Geobacillus* sp. strain G4 was grown in 10 mL of LB medium at 60°C for 48 hours (1). Cells were harvested by centrifugation at 8,000 × *g* for 15 minutes at 4°C. Genomic DNA was isolated using the Bacterial Genomic DNA Isolation Kit (Norgen Biotek Corp., Canada) according to the manufacturer’s instructions. The DNA libraries were prepared using the Illumina TruSeq Nano DNA Library Prep Kit (350), and sequencing was performed using an Illumina NovaSeq platform with paired-end reads, each 150 bases in length, by Macrogen Inc. (South Korea). The raw fastq files from the genome, consisting of 13,778,078 reads, were imported into the FastQC v0.12.1 tool for quality assessment (3). Quality filtering was performed using Trimmomatic v0.38. The remaining sequences were assembled using SPAdes v3.15.4. Short contigs (<200 bp) were removed from the assembly by seqtk v1.4 (https://github.com/lh3/seqtk). Assembly size and quality were evaluated using Quast v5.2.0 (4) and Busco v5.5.0 (5). Completeness and contamination parameters of the assembled genome were evaluated using CheckM v1.2.2 tool (6). NCBI Prokaryotic Genome Annotation Pipeline was used to annotate the genome using the RefSeq database (7). The 16S rRNA gene sequence of *Geobacillus* sp. strain G4 was extracted using barrnap v0.9 (https://github.com/tseemann/barrnap). Finally, for the identification of biosynthetic gene clusters related to secondary metabolism (BGCs) related to secondary metabolism in microorganisms, the AntiSMASH v7.1.0 software was utilized (8). Default parameters were used for all software tools unless otherwise noted.

The draft genome of *Geobacillus* sp. strain G4 was approximately 3.4 Mb with ~1,225× coverage. The assembly comprised 164 contigs with a G + C content of 52.59%. Genome completeness and contamination were 99.45% and 0.95%, respectively. The N50 and L50 values were 236,119 bp and 5 contigs, respectively. The annotation identified 3,490 genes: 2,047 coding sequences (CDS), 5 rRNAs, 88 tRNAs, 1 transfer-messenger RNA, and 1,349 putative proteins. Regarding gene clusters related to secondary metabolism (BGCs), five clusters were identified: betalactone, type III polyketide synthases, lanthipeptide-class-I, NI-siderophore, and terpene. The 16S rRNA gene sequence of *Geobacillus* sp. strain G4 showed a 99.87% identity with that of *Geobacillus kaustophilus* strain BGSC 90A1 (GenBank accession no. NR_115285.2) and a 99.80% identity with *Geobacillus thermoleovorans* strain LEH-1 (GenBank accession no. NR_036985.1). This genome enhances our understanding of thermophilic bacteria and their biotechnological potential.

## Data Availability

The Illumina raw reads were uploaded to the SRA database under the accession number SRR29836749. Genome assembly is available under the accession number ASM4032797v1. The draft genome sequence of *Geobacillus* sp. strain G4 has been deposited at GenBank under the accession number JBEPJY000000000, BioProject number PRJNA1124973, and BioSample number SAMN41878472.
